# Health rights knowledge among medical school students at King Abdulaziz University, Jeddah, Saudi Arabia

**DOI:** 10.1371/journal.pone.0176714

**Published:** 2017-05-01

**Authors:** Samia M. Al-Amoudi, Abdullah A. Al-Harbi, Nasser Y. Al-Sayegh, Basem S. Eldeek, Souzan M. Kafy, Mahmoud S. Al-Ahwal, Nabeel S. Bondagji

**Affiliations:** 1 Health Empowerment and Health Rights Unit, Faculty of Medicine, King Abdulaziz University, Jeddah, Saudi Arabia; 2 Sheikh Mohammed Hussein Al-Amoudi Center of Excellence in Breast Cancer, King Abdulaziz University, Jeddah, Saudi Arabia; 3 Faculty of Medicine, King Abdulaziz University, Jeddah, Saudi Arabia, Jeddah, Saudi Arabia; 4 Medical Education, Faculty of Medicine, King Abdulaziz University; 5 Faculty of Medicine, Mansoura University, Mansoura, Egypt; 6 Department of Obstetrics and Gynecology, Faculty of Medicine, King Abdulaziz University, Jeddah, Saudi Arabia; George Washington University, UNITED STATES

## Abstract

**Background:**

Health care is a basic human right, and Saudi Arabia affirms these rights for all its citizens.

**Objectives:**

To assess the knowledge of medical students regarding health rights in Saudi Arabia.

**Methods:**

This cross-sectional study was conducted at King Abdulaziz University (KAU) from September 2015 through November 2015. A questionnaire written in English collected demographic data and included questions about reproductive health care and health rights of women and patients with cancer, senility, or special needs.

**Results:**

Of the 267 participants, 184 (68.9%) were female, and 252 (94.4%) were Saudi. Regarding consent, 87 (32.6%) and 113 (42.3%) participants believed a female patient required the consent of a male guardian to receive medical treatment or surgery, respectively, in Saudi Arabia, and only 106 (39.7%) knew that a female patient could provide consent for a caesarean section. Sixty-six (24.7%) believed that abortion is never allowed in Islam. Only 93 (34.8%) were aware that acquired immunodeficiency syndrome (AIDS) and human immunodeficiency virus (HIV) patients had health rights, about half (144, 53.9%) knew that cancer patients have a right to full information, and most (181, 67.8%) believed that a patient had the right to withhold health information from his/her family. Approximately half were aware that cancer patients have the right to free medical treatment (138, 51.7%) or that health rights applied to special needs patients (137, 51.3%) and senile patients (122, 45.7%).

**Conclusions:**

The knowledge of KAU medical students regarding health rights of certain patient populations highlights the importance of health rights education in medical school.

## Introduction

Health care rights are basic human rights based on the concept of the fundamental dignity and equality of all human beings. The World Health Organization (WHO) acknowledges that the highest attainable standard of health is a fundamental right to every human being [1]. The Sustainable Development Goals include many goals and targets related to promoting health, gender equality, and the ability to make decisions about one’s own health [2]. The empowerment of women, and the community in general with knowledge about their health rights are essential to promote health. The right to health care is referred to in Article 31 of the Basic Law of Saudi Arabia, which declares, “the state takes care of health issues and provides health care for every citizen [3].” The Ministry of Health (MOH) issued the Patient’s Bill of Rights in 2006 [4]. The MOH affirms these health rights in its policies and procedures manual and through periodic circulars [5].

Medical students and health care providers have important roles in helping patients understand their health care rights, contributing to a safe and high quality health care system. They therefore need a good understanding of these rights to be able to uphold them. Unfortunately, in a study conducted by Alghanim, among 242 Saudi physicians and nurses, only 66.1% were aware of the MOH Patients’ Bill of Rights [6]. El-Sobokey et al., in a study at the College of Applied Medical Science at King Saud University, Riyadh, Saudi Arabia, found that only approximately one-quarter (23.4%) of the students stated that lecturers mentioned patient’s rights during teaching sessions [7]. The globalization of health care and movement of patients and health care providers around the world require that health care providers and students be introduced to these globally recognized rights as early as possible in their study and career. The objective of this study was to assess the current knowledge of King Abdulaziz University (KAU) medical students concerning health empowerment issues and health rights in Saudi Arabia.

## Methods

This cross-sectional study was conducted at KAU, Jeddah, Saudi Arabia from September 2015 through November 2015. Approval was obtained from the Unit of Biomedical Ethics, Research Ethics Committee at the Faculty of Medicine, KAU. The questionnaire was distributed to 300 medical students (years 4, 5, and the final year), and 267 responded with an 89% response percentage. The students were randomly selected from different years in medical school and from different surgical rotations. There was no coercion as all processes were supervised by senior faculty staff supervisors and the questionnaire was made anonymous. The objectives of the study were explained in detail to students at the College of Medicine, and a questionnaire written in English was distributed to students who agreed to participate. The questionnaire collected demographic data, and the students were asked to respond to questions about the following topics by answering “yes,” “no,” or “I don’t know”: reproductive health care practice; the rights of women to provide their own consent for medical treatment and procedures; and the health rights of cancer patients, the elderly, and special needs patients (deaf/mute).

To ensure content validity, the questionnaire was revised by four consultants at KAU Hospital and some questions were added. A pilot study was conducted among 30 students to improve validity. Some questions were deleted, others were modified. The questionnaires were collected, and the answers were analyzed.

### Statistical analyses

Statistical analysis was performed by a qualified statistician. Results are expressed as numbers and percentages. Significance was considered at P < 0.05. All data were qualitative, and the chi-square test was used. Statistical analyses were conducted using SPSS, version 22 (SPSS, Chicago, IL, USA).

## Results

Of the 267 medical students participating in this study, 184 (68.9%) were female, and 252 (94.4%) were Saudi. The mean age of participants was 19.5 years [standard deviation (SD), ± 2.0 years).

The results showed that most of the participants did not fully understand the health rights of women in Saudi Arabia (Table 1). For example, 91 (34.1%) and 89 (33.3%) of the participants believed that hospital rules require female patients to obtain a male guardian’s consent for hospital admission and discharge, respectively. Similarly, 87 (32.6%) and 113 (42.3%) believed that a male guardian was required to give consent for medical treatment and surgery, respectively, and only 102 (38.2%) and 69 (25.8%), respectively, correctly responded that the consent of a male guardian was not required, whereas 78 (29.2%) and 85 (31.8%), respectively, responded that they did not know. When asked specifically about a caesarean section, only 106 (39.7%) of the medical students were aware of a woman’s right to make this decision for herself and provide consent, whereas 39 (14.6%) believed that female patients did not have this right, and 122 (45.7%) did not know (Table 1).

**Table 1:** Medical students’ knowledge of the rights of female patients in Saudi Arabia.

Question	Yes		No		Don’t know	
	N	%	N	%	N	%
Do women in SA require a male guardian to: Obtain admission to a hospital	91	34.1	101	37.8	75	28.1
Be discharged from the hospital	89	33.3	86	32.2	92	34.5
To sign consent for medical treatment	87	32.6	102	38.2	78	29.2
To sign consent for surgery	113	42.3	69	25.8	85	31.8
In SA, can a woman consent for herself for a Caesarean section?	106	39.7	39	14.6	122	45.7

SA, Saudi Arabia; N, number.

Many participants also lacked knowledge about the health rights of elderly and disabled patients in Saudi Arabia. For example, only 116 (43.4%) of the participants were aware that the health rights law covers patients with disabilities, whereas 57 (21.3%) believed that this was not the case, and 94 (35.2%) responded that they did not know. About half of the participants (137, 51.3%) knew that special needs patients have health rights, whereas 63 (23.6%) did not believe this to be true. Similarly, 122 (45.7%) believed that the health rights law includes senile patients, whereas 50 (18.7%) did not (Table 2).

**Table 2:** Knowledge of medical students of the law and rights of patient with disabilities, special needs, and senility.

Question	Yes		No		Don’t know	
	N	%	N	%	N	%
Does the Saudi Arabian health rights law include: Those with disability?	116	43.4	57	21.3	94	35.2
Those with special needs?	137	51.3	63	23.6	67	25.1
Those with senility (elderly)?	122	45.7	50	18.7	95	35.6

N, number.

Regarding perceptions of reproductive health rights, approximately one-fourth (66, 24.7%) of the participants believed that abortion is completely forbidden in Islam, whereas half (134, 50.2%) were aware that abortion is allowed in special cases. When asked about a woman’s right to obtain contraception, 82 (30.7%) responded that consent from a male guardian/partner is first required, 42 (15.7%) responded that consent from a male guardian/partner was not required, and more than half (143, 53.6%) did not know. Another reproductive health right issue is premarital screening. Only 82 (30.7%) of the participants were aware that screening includes human immunodeficiency virus (HIV) testing, whereas most (164, 61.4%) responded that they did not know. When asked about HIV/acquired immunodeficiency syndrome (AIDS) patients in Saudi Arabia, 93 (34.8%) were aware that these patients have health rights, 50 (18.7%) thought that they do not have these rights, and 124 (46.4%) did not know. Concerning sexual/reproductive information, 88 (33.0%) were aware of this, 120 (44.9%) were not, and 59 (22.1%) did not know (Table 3).

**Table 3:** Medical students’ knowledge of reproductive health rights in Saudi Arabia.

Question	Yes		No		Don’t know	
	N	%	N	%	N	%
Abortion is never allowed in Islam?	66	24.7	134	50.2	67	25.1
Do they require a male guardian to obtain contraception for family planning?	82	30.7	42	15.7	143	53.6
In Saudi Arabia, does premarital screening include HIV testing?	82	30.7	21	7.9	164	61.4
Are there any rights for HIV/AIDS patients in Saudi Arabia?	93	34.8	50	18.7	124	46.4
Is sexual/reproductive information taught in Saudi Arabia?	88	33.0	120	44.9	59	22.1

N, number.

Responses to questions concerning the rights of cancer patients are shown in (Table 4). About half (144, 53.9%) of the participants believed that full disclosure of information pertaining to his/her illness is a patient right, and most (207, 77.5%) agreed that complete information should be provided to a newly diagnosed cancer patient about his/her disease. Most participants (181, 67.8%) also believed that a patient had the right to withhold information from his/her family, whereas 39 (14.6%) believed that the patient had no such right, and 47 (17.6%) responded that they did not know. About half (138, 51.7%) of the participants were aware that cancer patients in Saudi Arabia have the right to free medical treatment. However, fewer than half (115, 43.1%) knew that cancer patients have the right to free surgical treatment, whereas 39 (14.6%) believed cancer patients do not have this right, and 113 (42.3%) did not know (Table 4).

**Table 4:** Medical students’ knowledge of the health rights of cancer patients in Saudi Arabia.

Question	Yes		No		Don’t know	
	N	%	N	%	N	%
Is disclosure of full information one of the patient’s health rights?	144	53.9	48	18.0	75	28.1
Do you agree in providing full information to a newly diagnosed cancer patient about his/her disease?	207	77.5	32	12.0	28	10.5
Does the patient have the right to hide information from his family?	181	67.8	39	14.6	47	17.6
Do cancer patients in SA have the right to free:						
Medical treatment	138	51.7	31	11.6	98	36.7
Chemotherapy	131	49.1	31	11.6	105	39.3
Radiotherapy	125	46.8	26	9.7	116	43.4
Surgery	115	43.1	39	14.6	113	42.3

N, number.

A comparison of the responses of male and female medical students showed similar knowledge of women’s reproductive health rights (Tables 5 and 6).

**Table 5:** Male and Female medical student’s knowledge of the rights of female patients in Saudi Arabia^1^.

Question	Male			Female			*P* value
	Yes	No	Don’t know	Yes	No	Don’t know	
Do women in SA require a male guardian to:							
Obtain admission to a hospital	28 (33.7)	33 (39.8)	22 (26.5)	63 (34.2)	68 (37.0)	53 (28.8)	.891
Be discharged from the hospital	26 (31.3)	30 (36.1)	27 (32.5)	63 (34.2)	56 (30.4)	65 (35.3)	.652
To sign consent for medical treatment	30 (36.1)	29 (34.9)	24 (28.9)	57 (31.0)	73 (39.7)	54 (29.3)	.668
To sign consent for surgery	36 (43.4)	20 (24.1)	27 (32.5)	77 (41.8)	49 (26.6)	58 (31.5)	.909
In Saudi Arabia, can a woman consent for herself for a caesarean section?	35 (42.2)	12 (14.5)	36 (43.4)	71 (38.6)	27 (14.7)	86 (46.7)	.849

^1^Data are presented as frequency (percent) unless otherwise specified.

**Table 6:** Male and female medical students’ knowledge of reproductive health rights in Saudi Arabia^1^.

Question	Male			Female			*P* value
	Yes	No	Don’t know	Yes	No	Don’t know	
Abortion is never allowed in Islam?	26 (31.3)	33 (39.8)	24 (28.9)	40 (21.7)	101 (54.9)	43 (23.4)	.066
Do women in Saudi Arabia require a male guardian approval to obtain contraception for family planning?	28 (33.7)	11 (13.3)	44 (53.0)	54 (29.3)	31 (16.8)	99 (53.8)	.659
In Saudi Arabia, does premarital screening include HIV testing?	25 (30.1)	6 (7.2)	52 (62.7)	57 (31.0)	15 (8.2)	112 (60.9)	.949
Are there any rights for HIV/AIDS patients in Saudi Arabia?	29 (34.9)	19 (22.9)	35 (42.2)	64 (34.8)	31 (16.8)	89 (48.4)	.452
Is sexual/reproductive information taught in Saudi Arabia?	29 (34.9)	39 (47.0)	15 (18.1)	59 (32.1)	81 (44.0)	44 (23.9)	.566

Abbreviations: AIDS, acquired immunodeficiency syndrome; HIV, human immunodeficiency virus.

^1^Data are presented as frequency (percent) unless otherwise specified.

A larger proportion of male participants were aware that the Saudi Arabian health rights law covered all citizens (51.8% vs. 30.4%; *P* = 0.002), including patients with disabilities (55.4% vs. 38.0%; *P* = 0.016) or special needs (61.4% vs. 46.7%; *P* = 0.020) (Table 7).

**Table 7:** Male and female medical students’ knowledge of rights of patient with disability, special needs, and senility^1^.

Question	Male			Female			*P* value
	Yes	No	Don’t know	Yes	No	Don’t know	
Does the Saudi Arabian health rights law include:							
those with disability?	46 (55.4)	17 (20.5)	20 (24.1)	70 (38.0)	40 (21.7)	74 (40.2)	.016
those with special needs?	51 (61.4)	20 (24.1)	12 (14.5)	86 (46.7)	43 (23.4)	55 (29.9)	.020
those with senility (elderly)?	40 (48.2)	15 (18.1)	28 (33.7)	82 (44.6)	35 (19.0)	67 (36.4)	.857

^1^Data are presented as frequency (percent) unless otherwise specified.

Regarding the rights of cancer patients, a larger proportion of male medical students were aware of these patients’ right to free medical treatment (68.7% vs. 44.0%; *P* = 0.001), radiotherapy (59.0% vs. 41.3%; *P* = 0.012), and surgery (54.2% vs. 38.0%; *P* = 0.042). However, a larger proportion of female participants responded that these patients had a right to full disclosure of information (92.4% vs. 72.3%; *P* < .001) (Table 8).

**Table 8:** Male and female medical students’ knowledge of the health rights of cancer patients in Saudi Arabia^1^.

Question	Male			Female			*P* value
	Yes	No	Don’t know	Yes	No	Don’t know	
**Cancer patients in SA have the right to free:**							
Medical treatment	57 (68.7)	5 (6.0)	21 (25.3)	81 (44.0)	26 (14.1)	77 (41.8)	.001
Chemotherapy	46 (55.4)	12 (14.5)	25 (30.1)	85 (46.2)	19 (10.3)	80 (43.5)	.109
Radiotherapy	49 (59.0)	9 (10.8)	25 (30.1)	76 (41.3)	17 (9.2)	91 (49.5)	.012
Do you agree in providing full information to a newly diagnosed cancer patient about his/her disease?	58 (69.9)	8 (9.6)	17 (20.5)	149 (81.0)	24 (13.0)	11 (6.0)	.002
Is disclosure of full information one of the patient’s health rights?	46 (55.4)	14 (16.9)	23 (27.7)	98 (53.3)	34 (18.5)	52 (28.3)	.933
Does the patient have the right to hide information from his/her family?	53 (63.9)	14 (16.9)	16 (19.3)	128 (69.6)	25 (13.6)	31 (16.8)	.641

^1^Data are presented as frequency (percent) unless otherwise specified.

## Discussion

Health rights are basic human rights and constitute a cornerstone of patient treatment, as recognized by the WHO, international and national laws, as well as Islamic Sharia law. Therefore, the rights of individuals to make informed decisions concerning their own reproduction and health issues free of discrimination or coercion require protection and should be made clear to all involved (patients, health care providers, and the patient’s family). However, patients’ rights are not widely understood in Saudi Arabia, and many health care providers are unaware of the rules and regulations designed to protect these rights, which may result in suboptimal care. This situation was raised in the following statement concerning Saudi Arabia by the Convention on the Elimination of All Forms of Discrimination Against Women (CEDAW) Committee Report published in April 2008 [8]: “The committee expressed concern about the lack of information and data on health problems and expressed concern that women may require permission of their male guardian to access health facilities.” The CEDAW drew this conclusion because the Saudi doctors who were interviewed were ignorant of the rights of female patients to obtain health care without the permission of their male guardians.

Medical schools in Saudi Arabia do not teach their students about health rights, leaving them to learn about these rights on their own or during on-the-job training. Medical students constitute an important segment of health care providers. Giving them a complete understanding of existing laws and regulations concerning the health rights of all patient groups early in their education and training would enable them to empower their patients to take control of their own health care. This would help all citizens of Saudi Arabia enjoy the highest attainable standard of health, which is their fundamental right.

In this study, we assessed the knowledge of medical students in Saudi Arabia regarding reproductive health care rights, the rights of women to consent for their own medical treatments and procedures, and the rights of cancer patients, elderly patients with senility, and special needs patients. There is limited information about this topic in the literature, and few studies were available for comparison.

In our study, we found that the KAU medical students were not well informed about women’s health rights, reproductive health rights, or the rights of potentially vulnerable patients. Terminating a pregnancy is allowed in Islam under strict conditions: if continuation of the pregnancy would endanger the life or health of the expectant mother or if the fetus has major congenital abnormalities. This decision should take into consideration gestational age at the time of termination [9, 10]. The parents have the right to request termination if these criteria are fulfilled and they obtain approval of a committee consisting of three specialists. In our study, about half the medical students were not aware of this right.

Similarly, only about one-third of the students were aware that premarital screening in Saudi Arabia includes HIV testing or that HIV/AIDS patients have health rights in Saudi Arabia. It is not surprising that knowledge of rights pertaining to sexual health was particularly poor among these students, because discussion of this topic is discouraged in traditional Saudi society. Previous studies have also shown that the level of knowledge concerning sexual health is poor among adolescents and youth in Saudi Arabia [11, 12].

Regarding women’s health rights, about one-third of the medical students were not aware that female patients are allowed to admit themselves into the hospital and discharge themselves. What is more worrisome is that 42.3% of the students were not aware that a female patient has the right to provide her own consent for surgery according to the Saudi MOH rules and regulations [5]. The consequences of this lack of knowledge are seen in daily practice. A study that reviewed the consent forms of patients undergoing breast surgery in Saudi Arabia reported that for patients aged 40–49 years, 85% of the consent forms were signed by the patient herself, and for patients aged 50–59 years, 93% were signed by the patient herself. However, for the remaining patients, consent forms were signed by male guardians, suggesting that not all female patients are aware of their right to determine their course of treatment [13]. Failure to recognize that women can give consent for their own surgery can delay necessary and sometimes urgent procedures. In particular, a delayed caesarean section can lead to morbidity and mortality for both mother and fetus. Such an incident occurred in Saudi Arabia in 1984, when a mother died because of ignorance and ambiguity regarding these rules. This incident prompted the Senior Council of Ulema (scholars) to issue a resolution in the same year stating that an adult Muslim woman has the right to give consent for health care issues including a caesarean section [14]. Nevertheless, in our study conducted decades later, 45.7% of the medical students were not aware of this right.

The rights of cancer patients represent another important area, as disclosure of diagnosis or prognosis can present an ethical dilemma. Although medical ethics places a high value on providing truthful information to patients, disclosing bad news could lead to despair. In traditional societies such as Saudi Arabia, public attitude towards full disclosure is conservative, and cultural traditions encourage shielding the patient from devastating news, particularly when the patient is female. There is a misconception that the male guardian should be informed first, and that it is his decision whether to disclose the information to the female patient. The situation becomes more difficult when the family asks to hide the diagnosis from the patient, or the patient asks to hide information from the family. In our study, 53.9% of participants knew that disclosure of full information is a patient’s rights, which is similar to the responses of medical students in other studies [15, 16]. Most participants (67.8%) believed that the patient had the right to hide this information from his/her family, whereas 14% believed that the patient had no such right.

The health rights of patients with special needs are of critical importance, and few studies have investigated this issue. For example, deaf patients rarely receive full information about their health care and rights, because sign language is not used in clinical practice in Saudi Arabia [17]. Many of our study participants did not understand the health rights of patients with special needs or senility in Saudi Arabia, highlighting the vulnerability of this patient population, which needs special care and attention.

## Conclusions

Medical students in Saudi Arabia have a poor understanding of health rights supported by Sharia law (i.e., Islamic law) and the rules and regulations set forth by the Saudi MOH. As future doctors, knowledge of the existing laws and regulations concerning the health rights of all patient groups is needed to deliver optimal health care. We therefore recommend including the subject of patient health rights in the curricula of schools of medicine, nursing, pharmacy, and other health fields.

## Supporting information

S1 Table Questionnaire used to survey students. (DOCX)
