# Supplementary material for: Health rights knowledge among medical school students at King Abdulaziz University, Jeddah, Saudi Arabia
Source: PLoS One. 2017 May 1;12(5):e0176714. doi: 10.1371/journal.pone.0176714 (PMC5411029; doi:10.1371/journal.pone.0176714)
Supplement: S1 Table — (DOCX) [file pone.0176714.s001.docx]

Health Rights Knowledge among Medical School Students at King Abdulaziz University, Jeddah, Saudi Arabia.

Dear student, kindly fill the questionnaire, the objective of this is to assess your current knowledge about health rights in Saudi Arabia. The survey will take only a few minutes to complete. All your answers will be kept private and anonymous. Nobody will be able to identify your answers.

| DEMOGRAPHIC DATA | | | | | | | | | | |
| --- | --- | --- | --- | --- | --- | --- | --- | --- | --- | --- |
| Name | |  | | Age |  | 🞏 Male | | 🞏 Female | | |
| Nationality | | 🞏 Saudi | 🞏 Non-Saudi | | | | | | | |
|  | GENERAL | | | | | | Yes | | No | Don’t know |
| 1. | Do they require a male guardian to: | | | | | |  | |  |  |
|  | 1. Obtain admission to a hospital | | | | | | 🞏 | | 🞏 | 🞏 |
|  | 1. Be discharged from the hospital | | | | | | 🞏 | | 🞏 | 🞏 |
|  | 1. To sign consent for medical treatment | | | | | | 🞏 | | 🞏 | 🞏 |
|  | 1. To sign consent for surgery | | | | | | 🞏 | | 🞏 | 🞏 |
|  | 1. Can a woman consent for herself for a caesarean section? | | | | | | 🞏 | | 🞏 | 🞏 |
| 2. | Does the Saudi Arabian health rights law include: | | | | | |  | |  |  |
|  | 1. those with disability? | | | | | | 🞏 | | 🞏 | 🞏 |
|  | 1. those with special needs? | | | | | | 🞏 | | 🞏 | 🞏 |
|  | 1. those with senility (elderly)? | | | | | | 🞏 | | 🞏 | 🞏 |
|  | REPRODUCTIVE | | | | | | Yes | | No | Don’t know |
| 3. | Abortion is never allowed in Islam? | | | | | | 🞏 | | 🞏 | 🞏 |
| 4. | Are there any rights for HIV/AIDS patients in Saudi Arabia? | | | | | | 🞏 | | 🞏 | 🞏 |
| 5. | Do they require a male guardian to obtain contraception for family planning? | | | | | | 🞏 | | 🞏 | 🞏 |
| 6. | In Saudi Arabia, does premarital screening include HIV testing? | | | | | | 🞏 | | 🞏 | 🞏 |
| 7. | Is sexual reproductive information taught in Saudi Arabia? | | | | | | 🞏 | | 🞏 | 🞏 |
|  | CANCER | | | | | | Yes | | No | Don’t know |
| 8. | Cancer patients in Saudi Arabia have the right to free: | | | | | |  | |  |  |
|  | 1. Medical treatment | | | | | | 🞏 | | 🞏 | 🞏 |
|  | 1. Chemotherapy | | | | | | 🞏 | | 🞏 | 🞏 |
|  | 1. Radiotherapy | | | | | | 🞏 | | 🞏 | 🞏 |
|  | 1. Surgery | | | | | | 🞏 | | 🞏 | 🞏 |
| 9. | Do you agree in providing full information to a newly diagnosed cancer patient about his/her disease? | | | | | | 🞏 | | 🞏 | 🞏 |
| 10. | Is disclosure of full information one of the patient’s health rights? | | | | | | 🞏 | | 🞏 | 🞏 |
| 11. | Does the patient have the right to hide information from his/her family? | | | | | | 🞏 | | 🞏 | 🞏 |

*Thank you*
